# The history of diapers and their environmental impact

**DOI:** 10.1038/s41390-024-03347-5

**Published:** 2024-07-09

**Authors:** Cansu Tokat, Nicholas C. Rickman, Cynthia F. Bearer

**Affiliations:** 1 Department of Pediatrics, Case Western Reserve University School of Medicine, Cleveland, OH 44106 USA; 2 Division of Neonatology, Department of Pediatrics, UH Rainbow Babies & Children’s Hospital, Cleveland, OH 44106 USA

## Abstract

- This article examines diaper practices around the world throughout history.
- This article reviews the innovation of the modern diaper and the environmental effects of disposable diapers.

## The history and the future of diapers

The story of diapers is as old as humans. The materials and methods used for diapering have varied throughout history, depending on people’s traditions, cultures, and geographic locations. It was not until the early twentieth century that disposable diapers were invented. These inventions led to the rapid development of prototypes for the modern diapers we still use today.

### Once upon a time, diapers

**Fig. 1:**
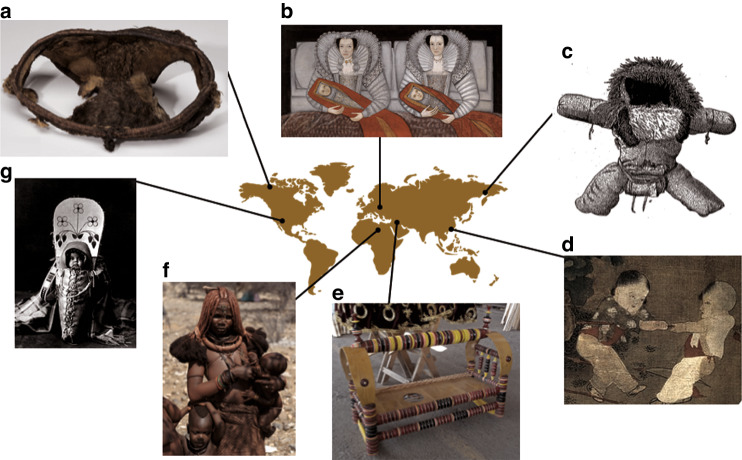
Different diaper practices around the world throughout history (Figured made with Biorender). **a** An eskimo diaper made of seal skin, found in Point Barrow, Alaska. Dated to 800–1000 AD (https://nmnh.typepad.com/rogers_archaeology_lab/2014/03/sealskinbabypants.html). **b** Swaddling dates from as early at 4000–4500 BC but fell out of favor in the 17th century. Depicted here are the Cholmondeley Ladies and their swaddled babies. **c** 1600–1610 (https://en.wikipedia.org/wiki/Swaddling). **c** Chuckchi of the northernmost eastern portions of Russia (approximately 500 BC – present) made fur infant dresses with a square diaper sewn on the back (https://nmnh.typepad.com/rogers_archaeology_lab/2014/03/sealskinbabypants.html). **d** Chinese open crotch pants were a traditional method of toileting training children, still in use mainly in rural China today (prehistoric – present) (https://www.nspirement.com/2017/07/13/paintings-of-children-by-master-su-hanchen.html). **e** Cradleboards with built-in holes for excreta and “urine tubes” for waste containment were used by the Kurdish (prehistoric – present) (https://www.greenprophet.com/2012/04/uzbeki-babies-diapers/). **f** Several African cultures practice skin to skin contact and elimination communication, obviating the need for diapers (prehistoric – present) (https://www.randafricanart.com/African_maternity_figures_main_page.html). **g** Cradleboards still in use in some indigenous cultures are used for carrying the infant and excreta containment (prehistoric – present) (https://native-american-totems.com/sacred-path-medicine/the-cradleboard-on-your-sacred-path/attachment/cradleboard/).

The oldest known diaper use was in prehistoric cultures in cold climates (Fig. 1). The Chukchi society used animal skins as diapers for insulation in colder climates, such as Siberia. The babies were placed in fur bags with four extra bags for their arms and legs and a big flap between their legs. Dry moss was filled in the flap, which was changed whenever the baby cried. The Inuit people in northern North America used moss and sealskin as diapers. In addition to keeping their babies dry, moss and sealskin kept them warm. The mothers would change their babies when they were warm inside their dwellings.^1^

Prehistorically, the Navajo people used cradleboard called “Aweetsaal”, literally meaning “baby diaper”, for containment of excreta as well as for carrying the infant on the back of the mother or other family member. The bark of dessert cliffrose was shredded and placed under the baby in the cradleboard.^2^

Another unique solution was reported for a large group of ancient societies in Central Asia, where some cradleboards had built-in “urine tubes” for waste containment. Infants were tightly swaddled to the cradle to maintain immobility. A reed tube was attached to the child’s genitalia (A reed tube had a circular opening for males and an oval opening for female babies.) The end of the tube was fitted into a hole in the cradle’s base. It was necessary to place a pot under the cradle to collect urine.^3^

In the age old Anatolia region of Turkey, some mothers placed boiled clay (Höllük; “boiled clay” is the operation of sifting and drying clayed soil and putting it under the baby) under their baby’s bottoms to keep them dry and warm.^4^

Swaddling began around 4000 BC and was Europe’s most common diapering method. It didn’t change much until the Industrial Revolution. The babies were swaddled with their excreta in cotton or linen strips for at least three days without changing (https://en.wikipedia.org/wiki/Swaddling).

In the Japanese culture starting in the Edo period (1603–1867), parents used their well-worn kimonos, which were cut in a diaper shape, in order to contain excreta and provide better protection for the baby.^5^

Historically, diapers were not commonly used in humid and warm climates. In many cultures, mothers used what is now known as elimination communication. Several age old African cultures practiced skin-to-skin contact, so mothers could quickly detect signs and signals of their babies’ toileting needs. When they signaled, they would hold their babies over a bucket or out in the street while the babies would eliminate their waste.^5^

Until recently, Chinese mothers would feel their baby’s peristaltic movement while breastfeeding and be able to hold the newborn away from themselves for the baby to have a bowel movement. Young children had slits made in their underpants or shorts (Kāidāngkù; open crutch pants), allowing excretion while avoiding soilage of clothing (https://en.wikipedia.org/wiki/Open-crotch_pants).

The Vietnamese tradition used diapers rarely. Parents used a whistling sound at certain times to remind their children to eliminate. Parents would frequently check their babies for signs of their need to eliminate.^6^

### The safety pin and the cloth diaper

By the 1880s, the safety pin had been invented by Walter Hunt, which helped create tighter protection around the baby’s bottom to prevent leakage (https://lemelson.mit.edu/resources/walter-hunt).

Obtaining diapers, easily available today, was one of the biggest problems in childcare. In the late 19th and early 20th century, cloth diapers were among the most common choices. A common practice was to make diapers out of old cloth and then cover them with tighter knitting and loose, ill-fitting plastic pants to prevent soiling the babies’ clothes. However, these diapers produced irritant diaper dermatitis, causing infants to be very uncomfortable. In addition, diapers were washed at home in hot water using harsh chemicals that also caused irritant dermatitis.^7^ Of historical interest, the preterm babies in at least one Baby Incubator Exhibit of the late 1800’s early 1900’s (Alaska-Yukon-Pacific Exposition (1909) Baby Incubator Exhibit and Café) wore these cloth diapers (https://www.historylink.org/File/8921).

As women joined the workforce alongside men during World War II, they had neither the time nor energy to wash diapers at home after work. This change brought the necessity of diaper laundry services. Diaper laundry services delivered clean, sterilized diapers to homes and collected dirty diapers from homes each week. Diaper laundry services divided diaper washing into eleven cycles with detergent, followed by four cycles with boiling water only, reducing the exposure of babies to harsh chemicals.^5^

### The disposable diaper

The 1940s brought with it the innovation of disposable diapers. With Paulistróm Bruk from Sweden in 1942 and Marion Donovan from the United States in 1946, the era of disposable diapers started. The first disposable diapers by Paulistróm were designed using cellulose sheets because cotton was unavailable in Sweden during World War II. This diaper was a cellulose cloth held in place by rubber underpants. Meanwhile, in the United States, Marion Donovan invented the plastic cover to prevent the leakage of liquids. She used a shower curtain and designed the first leak-proof diaper cover at her sewing machine. She called her design the “Boater” because it helped babies “stay afloat.”^5^(https://www.historylink.org/File/8921).

Victor Mills, an American chemical engineer, launched Pampers in 1961. He used a disposable cellulose pulp core in a disposable diaper. It wasn’t until the 1970s that lateral tabs were first introduced as fasteners for diapers. They were first made with Velcro, then with a plastic material that could be opened and closed repeatedly. Over the years, diapers have evolved to provide more effective absorbing capabilities. More ergonomic, hourglass shape diapers replaced the thick rectangular diapers (https://lemelson.mit.edu/resources/marion-donovan).

### Modern disposable diaper

In the early 1960s, the United States Department of Agriculture was working on materials to improve water conservation in soil. They invented a super absorbent material, acrylonitrile polymer, which can absorb water more than 100 times its weight. In addition, the gel did not release liquid water like fiber-based absorbents.^8^ Following this discovery, Frank Carlyle Harmon and Billy Gene Harper made the production of modern “superabsorbent” disposable diapers possible (https://en.wikipedia.org/wiki/Superabsorbent polymer). As a result of these innovations, a better diaper was created, and these improvements have significantly reduced diaper dermatitis.

### Diapers nowadays

For the time being, disposable diapers are essential for baby care almost all around the world. An average child wearing diapers for the first 2.5 years will use between 6000 and 7000 disposable diapers. There have been a number of issues associated with disposable diapers as they have become more prevalent (https://lemelson.mit.edu/resources/marion-donovan).

Diaper dermatitis is still one of the most common skin disorders affecting between 7 and 35 percent of infants at least once, and it is caused by various factors (chemical, physical, enzymatic, microbiological), not solely diapers (https://en.wikipedia.org/wiki/Carlyle Harmon). Through good hygiene practices and regular diaper changes, diaper rashes can be reduced, but not eliminated.

At this point in time, diaper disposal has both health and environmental consequences. There is no doubt that disposable diapers contribute significantly to landfill waste. The Environmental Protection Agency estimates that single-use diapers are the third most common consumer item in landfills in the USA, and they take 500 years to biodegrade. As a result of these issues, both scientists and diaper companies are working to produce more environmentally friendly diapers.

The research studies over the past years showed that reducing the amount of material used in diaper production can lessen the negative impact on the environment. Novel designs to reduce the amount of materials in diapers result in disposable diapers that are more cost-efficient and environmentally safe.^9^

**Fig. 2:**
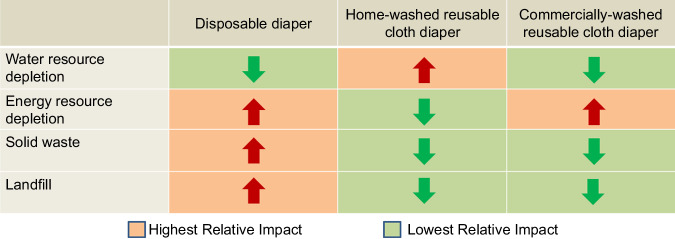
Table showing the impact of various types of diapers on the use of water and energy, the amount of solid waste, and size of landfills.

Because of the adverse environmental effect of disposable diapers, people started to show interest in reusable cloth diapers. Even if cloth diapers have the less environmental effect, each choice brings different problems. (Fig. 2) Nevertheless, disposable diapers produce 20 times more solid waste than reusable diapers, and disposable diapers have the highest impact on landfill. The consumer has more control over the environmental impact of home-washed reusable cloth diapers; recycling water and using renewable electricity will reduce the impact of home-washed reusable cloth diapers. Also, commercially washed reusable cloth diapers are another option.^10^ Novel processes for the recycling of diapers have good potential for decreasing the disposal impacts of disposable diapers. Studies on two pilot recycling plants, in the United Kingdom and Italy, showed that recycling disposable diapers decreases their life cycle environmental impact across all impact categories relative to current disposal practices. Another recycling plant from Taiwan specializes in diaper recycling for institutions - like long-term care facilities, or hospitals - to give old diapers new life. They use an on-site washing machine to sanitize used diapers so diaper waste can be processed into reusable raw materials. The machine can process up to 220 pounds of soiled diapers into clean, raw materials in just one hour. More studies are being conducted on recycling diaper waste, and several products use more biodegradable materials.

In addition to these diaper recycling efforts, researchers in Indonesia have focused on recycling disposable diaper waste as composite material for a structural and architectural component of the building for low-cost housing. A pilot project from the United Kingdom uses sanitized diaper waste as a road paving material to build highways. Repurposing disposable diaper waste into construction materials and highways could give disposable diapers more options for further use; therefore, disposable diapers would be less damaging to the environment.^10–12^ (https://inhabitat.com/this-groundbreaking-new-machine-can-recycle-220-pounds-of-diapers-in-a-single-hour/) (https://www.washingtonpost.com/climate-solutions/2022/02/18/diaper-highway-nappy-recycling/).

However, with the devastating effect of disposable diapers on the environment, we still have a long way to go to find the best solution for our babies and our environment.

In conclusion, the modern diaper was developed from the collective perseverance of humankind and the combination of innovation and cutting-edge scientific advancement. Nevertheless, further research is needed to end the environmental impact of diaper waste and eliminate toxic materials used in disposable diaper production.
